# Development, Analytical Characterization, and Bioactivity Evaluation of *Boswellia serrata* Extract-Layered Double Hydroxide Hybrid Composites

**DOI:** 10.3390/molecules28186449

**Published:** 2023-09-05

**Authors:** Stefania Cometa, Francesco Busto, Andrea Castellaneta, Andrea Cochis, Ziba Najmi, Rosanna Rizzi, Ilario Losito, Elvira De Giglio

**Affiliations:** 1 Jaber Innovation s.r.l., Via Calcutta 8, 00144 Rome, Italy; stefania.cometa@jaber.it; 2 Department of Chemistry, University of Bari, Via Orabona 4, 70126 Bari, Italy; f.busto3@studenti.uniba.it (F.B.); andrea.castellaneta@uniba.it (A.C.); ilario.losito@uniba.it (I.L.); 3 INSTM, National Interuniversity Consortium of Materials Science and Technology, Via G. Giusti 9, 50121 Florence, Italy; 4 Center for Translational Research on Autoimmune and Allergic Disease, CAAD, Department of Health Sciences, Università del Piemonte Orientale UPO, 28100 Novara, Italy; andrea.cochis@med.uniupo.it (A.C.); ziba.najmi@uniupo.it (Z.N.); 5 Institute of Crystallography, National Research Council (CNR), Via G. Amendola, 122/o, 70126 Bari, Italy; rosanna.rizzi@ic.cnr.it; 6 SMART Inter-Department Research Center, University of Bari, Via Orabona 4, 70126 Bari, Italy

**Keywords:** *Boswellia serrata* extract, layered double hydroxides, composite, drug delivery system, antimicrobial, anti-inflammatory

## Abstract

*Boswellia serrata Roxb.* extract (BSE), rich in boswellic acids, is well known as a potent anti-inflammatory natural drug. However, due to its limited aqueous solubility, BSE inclusion into an appropriate carrier, capable of improving its release in the biological target, would be highly desirable. Starting with this requirement, new hybrid composites based on the inclusion of BSE in a lamellar solid layered double hydroxide (LDH), i.e., magnesium aluminum carbonate, were developed and characterized in the present work. The adopted LDH exhibited a layered crystal structure, comprising positively charged hydroxide layers and interlayers composed of carbonate anions and water molecules; thus, it was expected to embed negatively charged boswellic acids. In the present case, a calcination process was also adopted on the LDH to increase organic acid loading, based on the replacement of the original inorganic anions. An accurate investigation was carried out by TGA, PXRD, FT-IR/ATR, XPS, SEM, and LC-MS to ascertain the nature, interaction, and quantification of the active molecules of the vegetal extract loaded in the developed hybrid materials. As a result, the significant disruption of the original layered structure was observed in the LDH subjected to calcination (LDHc), and this material was able to include a higher amount of organic acids when its composite with BSE was prepared. However, in vitro tests on the composites’ bioactivity, expressed in terms of antimicrobial and anti-inflammatory activity, evidenced LDH–BSE as a better material compared to BSE and to LDHc–BSE, thus suggesting that, although the embedded organic acid amount was lower, they could be more available since they were not firmly bound to the clay. The composite was able to significantly decrease the number of viable pathogens such as *Escherichia coli* and *Staphylococcus aureus,* as well as the internalization of toxic active species into human cells imposing oxidative stress, in comparison to the BSE.

## 1. Introduction

BSE, an oleogum resin of Indian frankincense, mainly employed in traditional Asiatic medicine, has recently gained growing popularity in the phytochemical-based management of a variety of inflammatory diseases (such as arthritis, osteoarthritis, inflammatory bowel disease, allergy, and asthma) [1,2]. In addition, recent studies have evidenced also the potential antimicrobial activity of the extract [3,4]. The main active compounds contained in BSE are pentacyclic triterpenic acids, called boswellic acids (BAs), which are considered the main agents responsible for the anti-inflammatory activity of the resin. The main drawback in the use of BSE is related to its poor aqueous solubility and limited bioavailability. In this respect, different inclusion strategies have been proposed to enhance its solubility, dissolution rate, and body absorption, mainly based on the employment of phospholipids, cyclodextrins, or, in general, polymer-based vehicles or emulsions [5,6,7,8].Recently, many delivery vehicles for pentacyclic triterpenes, including microemulsions [9], nanoparticles [10,11], nanomicelles [12], and solid lipid nanoparticles (SLNs) [13], have been developed to solve the poor aqueous solubility and absorption. Nevertheless, these vehicles could provide a low loading capacity, high costs, and potential safety concerns. In the present study, to overcome these disadvantages, a surfactant-free strategy has been proposed to efficiently load and deliver BSE components using a clay as a carrier.

Nowadays, extensive research efforts in pharmaceutical and biopharmaceutical science have been addressed to the possibility to embed active ingredients in clays, such as lamellar host lattices, as alternatives to polymeric carriers, to develop inorganic–organic hybrids [14,15,16]. These lamellar clays aim to enable the drug’s release in its pharmaceutical target, modify the drug’s pharmacokinetics or release rate—for example, by maintaining pharmacologically active drug levels for long periods and thus avoiding repeated administrations—or to enhance the drug bioavailability, a problem that is particularly frequent in the case of natural extracts containing different lipophilic molecules, such as BSE.

In this scenario, a particularly appealing candidate for the development of these hybrid composites is represented by layered double hydroxides (LDHs), which are based on positively charged brucite-like layers with divalent and trivalent cations coordinated with hydroxyl groups, balanced by the anions in the interlayer spaces [17]. These clays, present in nature, can be also easily synthesized in the laboratory by means, among others, of three main synthetic preparation roots, i.e., (1) an exchange reaction, (2) direct coprecipitation, and (3) hydrothermal methods [18]. Regarding their toxicity, LDHs were found to show minimal cytotoxic effects [19]. Moreover, LDHs are already used in medicine for their antiacid and antipepsin activity [17]. Due to their interlayer cages filled with different charge-balancing anions (usually nitrate or carbonate anions), LDHs can be applied to encapsulate various organic guests having an anionic nature. LDHs containing carbonate as an interlayer anion are not particularly prone to anion exchange but, if thermally treated, they can be decomposed into a mixed oxide and then, in this form, they can re-adsorb water and anions as they return to their original structure. Therefore, calcined hydrotalcites can be a very interesting support material for the intercalation of organic molecules [20].

In this study, we have developed for the first time an LDH–BSE inorganic–organic hybrid composite, with the aim to obtain a supramolecular structure able to enhance the anti-inflammatory and antimicrobial abilities of BSE. Different works have documented the advantages of employing LDHs to carry poorly soluble molecules [21,22,23]. Here, we have chosen a magnesium aluminum carbonate, as received (LDH) or thermally treated by calcination at 450 °C (LDHc), to embed a BSE rich in BAs. The chemical composition, in terms of abundance percentages, of the different BAs in the extract, as well as when embedded in the LDH and LDHc carriers, was investigated by means of liquid chromatography–mass spectrometry (LC-MS) analysis. The hybrid composites were analytically characterized by Fourier transform infrared spectroscopy (FT-IR), powder X-ray diffraction (PXRD), X-ray photoelectron spectroscopy (XPS), scanning electron microscopy (SEM), and thermogravimetric analysis (TGA).

## 2. Results and Discussion

### 2.1. Thermal Analysis

**Figure 1:**
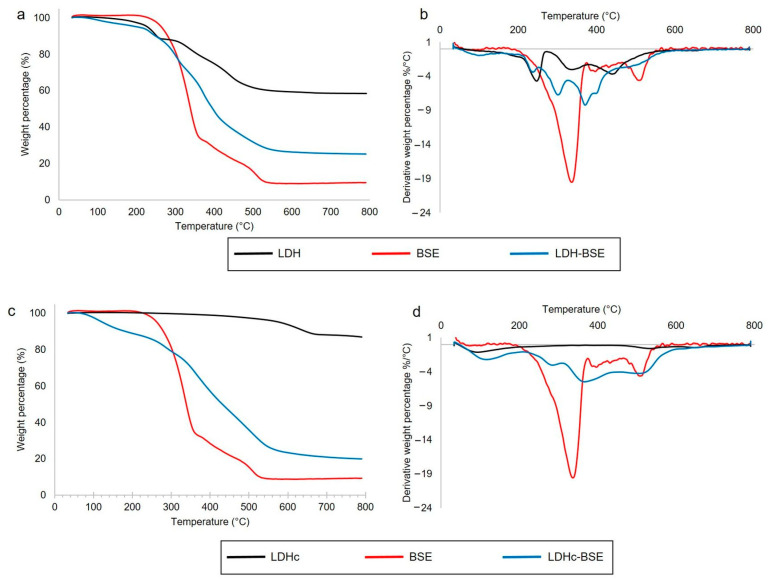
TG (**a**,**c**) and derivative TG (**b**,**d**) traces in the range 30–800 °C in nitrogen of (**a**,**b**) LDH, BSE and LDH–BSE; (**c**,**d**) LDHc, BSE, and LDHc–BSE.

The thermal properties of the inorganic–organic composites were studied by means of thermogravimetric analysis (TGA). In Figure 1, the thermogravimetric (TG) (a,c) and derivative thermogravimetric (DTG) traces (b,d) of LDH- and LDHc-based hybrids, as well as their feed materials, are reported.

The TG and DTG diagrams of the LDH (black line, panels a and b, respectively) showed an initial weight loss of up to 180 °C, equal to 2%, corresponding to the desorption of surface water. The second weight loss, which occurred from 180 to 270 °C, was approximately 9% and corresponded to the desorption of interlayer water, with a peak at 248 °C. The third and fourth weight losses occurred at 340 and 440 °C (15 and 16% of weight loss, respectively), related to the decomposition of carbonate anions (with CO_2_ evolution) and to the dehydroxylation of the mixed metal oxides. Similar results were reported by other authors [20,24]. The residue at 800 °C was equal to 58%. Stanimirova et al. reported that hydrotalcite thermal decomposition passed through a series of metaphases, forming a mixture of MgO and MgAl_2_O_4_ as the final product of thermolysis [25].

The TG and DTG diagrams of the LDHc (black line, panels c and d, respectively) showed curves that were completely different from those relevant to the non-calcined sample. Indeed, excluding the initial step due to surface water evolution (up to 180 °C, corresponding to 5%), thermal decomposition of the calcined sample occurred at very high temperatures, i.e., in the range 460–700 °C, with 7% of weight loss. The residue at 800 °C was equal to 88%. These findings confirmed the occurred calcination.

BSE thermogravimetric analysis (red line, present in all the panels) showed negligible water/volatile content and a main thermal event occurring in the range 200–365 °C, with a sharp peak at 339 °C, corresponding to a 66.5% weight loss percentage, followed by several minor thermal events in the range 365–545 °C, corresponding to a 24.5% weight loss percentage. The residue at 800 °C was equal to approximately 9%.

**Figure 2:**
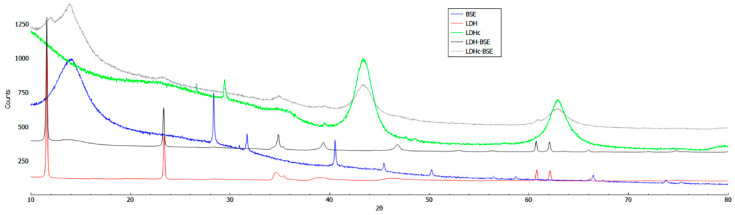
From top to bottom, the powder X-ray diffraction patterns of LDHc–BSE, LDHc, BSE, LDH–BSE, and LDH.

Finally, the thermogravimetric curves of the BSE-embedded LDH materials (see blue lines in Figure 2) revealed a multi-step decomposition pattern. Especially in the case of the LDH, due to the initial complexity of the TG and DTG traces related to the clay, a strong overlap between these mass loss events and those relevant to BSE was here observed, making any single event difficult to disentangle. At 800 °C, a residue of 25 and 21% was found for LDH–BSE and LDHc–BSE specimens. These values, apparently similar, must be related to the residues of the bare LDH and LDHc samples: considering that the residue at 800 °C of the LDHc was significantly higher than that relevant to the LDH (i.e., 88 vs. 58%), it was hypothesized that a huge increment in BSE loading occurred in the calcined clay. This observation was confirmed by LC-MS analysis (*vide infra*). Finally, the TGA results indicated that both LDH and LDHc embedding BSE were thermally stable up to approximately 200 °C.

### 2.2. Powder X-ray Diffraction Analysis

PXRD is a powerful tool commonly used for hydrotalcite compounds to verify the expansion occurring between the hydroxide layers after the substitution of relatively small divalent anions, such as sulfate, nitrate, or carbonate, with bulky molecules. This expansion serves as crucial evidence of intercalation inside the lamellar host structure.

In Figure 2, the PXRD patterns of LDH–BSE and LDHc–BSE, as well as the corresponding patterns of the feed materials (*Boswellia serrata* powder extract, LDH, and LDHc), are compared. The experimental LDH pattern exhibited well-defined and characteristic peaks, which could be indexed by a hexagonal lattice with R-3m rhombohedral symmetry, which is commonly used to describe LDH structures [26]. In addition, the two strongest and most characteristic low-angle reflections, 003 and 006, were observed approximately at 11.6° (2ϑ) (d = 7.6 Å) and 23.4° (2ϑ) (d = 3.8 Å), respectively, with values closely aligned with the reference pattern for the carbonated LDH phase, thus indicating a well-crystallized sample.

In the LDH structure, the unit cell parameters, a and c, represent the average distance between two metal ions in the layers and three times the distance from the center of one layer to the next, respectively. The value of a (=2d110) depends on the average radii of the metal cations, while the value of c (=3d003) depends on their average charge, the nature of the interlayer anion, and the water content [27,28].

In contrast, the LDHc exhibited a significantly different pattern, where the peaks corresponding to the 003 and 006 reflections disappeared, indicating the complete destruction of the layered structure of the original hydrotalcite.

When the LDHc was exposed to the BSE, it partially reacquired the original layered structure, as shown in Figure 2. The LDH structure, even when calcined, can accommodate different types of anions/molecules and, due to the different sizes of the counterions, variations in the lattice parameters are expected, resulting in peak positions shift [29].

In our study, the PXRD patterns of the resulting products (LDH–BSE and LDHc–BSE) retained the major characteristic features of the corresponding LDH and LDHc, with no shift in the peaks towards lower angles. The unaltered basal spacing (d003) clearly indicated that the intercalation of BSE acid anions within the lamellar host structure did not occur in the composite organic–inorganic derivatives.

### 2.3. Bulk and Surface Investigations

**Figure 3:**
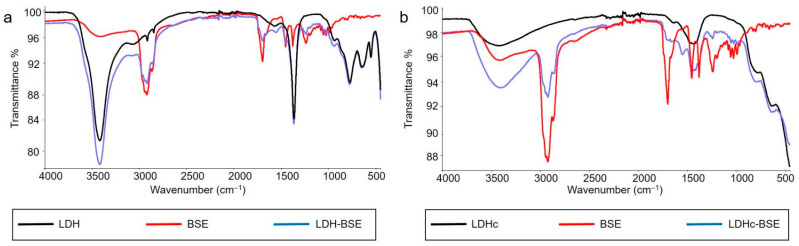
FT-IR/ATR spectra of (**a**) LDH, BSE, and LDH–BSE and (**b**) LDHc, BSE, and LDHc–BSE.

FT-IR/ATR analysis was carried out on the hybrid composites as well as on their feed materials to highlight possible organic–inorganic moiety interactions. In Figure 3, the ATR spectra of the LDH, BSE, and LDH–BSE (panel a) and LDHc, BSE, and LDHc–BSE (panel b) are reported.

Regarding the pristine LDH sample, a broad band at approximately 3419 cm^−1^ was due to the stretching of hydroxyl groups of water and hydroxyl groups located between the layers [30], while the peak at 1366 cm^−1^ could be attributed to carbonate [20]. The other peaks under 800 cm^−1^ could be assigned to metal–oxygen and metal hydroxide stretching modes.

The ATR spectrum of BSE revealed the presence of absorption bands at 3423 cm^−1^ (OH stretching), 2924 cm^−1^ (C-H stretching), 1699 cm^−1^ (C=O stretching of aryl acid), 1454 cm^−1^ (C=C bending), and 1241 cm^−1^ (C=C-C=O stretching of aryl ketone) [6].

No changes in the LDH–BSE ATR spectrum were observed with respect to those of the starting materials. Indeed, the spectrum appeared as a superimposition of the LDH and BSE spectra, thus suggesting simple BSE absorption on the clay.

In the LDHc sample, the –OH stretching band intensity significantly decreased after calcination, due to the water and interlayer hydroxyl groups’ loss caused by calcination. In the wavenumber range 1500–1300 cm^−1^, peaks assigned to residual interlayer CO_3_^2−^ anion were detected, as reported by Mališová and coworkers, who found a total peak disappearance at calcination temperatures higher than 550 °C [31]. The same authors stated that, in the calcination process, overheating must be absolutely avoided, since it could lead to a spinel structure resistant to rehydration, thus not allowing the reconstruction of a hydrotalcite-like structure where the anions of interest can be hosted. In this respect, the calcination temperature chosen in this work was maintained at 450 °C.

Finally, the LDHc–BSE spectrum showed a decrease in the vibration band at 1699 cm^−1^ due to the C=O bond present in the carboxyl group of BAs, in addition to the appearance of the bands at 1549 and 1379 cm^−1^ due to the asymmetric and symmetric stretching vibrations of carboxylates. This means that some of the BAs were stored in the LDH in their ionic form and probably established ionic interactions with the clay, which in turn could explain the partial BA extraction by methanol evidenced in the LC-MS studies (*vide infra*).

XPS analysis was carried out to obtain information on the surface compositions of the developed hybrid composites and to highlight possible clay–active principle interactions. In Table 1, the surface compositions of the composites, as well as the feed materials, are reported.

It can be observed that the surface composition of the hydrotalcite-based materials, in terms of Mg/Al ratio, does not reflect the stoichiometric one, typical of MgAl carbonate clays. This phenomenon is not surprising, since the chemistry of the surfaces often differs from that of the bulk [32]. Indeed, an Mg/Al ratio equal to 0.7:1, 0.7:1, 1.4:1, and 0.8:1 was recorded for the LDH, LDH–BSE, LDHc, and LDHc–BSE, respectively. Another important finding was the decreased carbon amount after the calcination process; on the other hand, a significant increase in the C1s atomic percentage was detected after BSE embedding, both on the pure and calcined hydrotalcite. Considering, for example, the C1s/Al2p ratio, this ratio changed from 1.5:1 to 49:1 when the LDHc was loaded with BSE (i.e., approximately 33 times higher) and from 9:1 to 51:1 in the case of the LDH-based system (i.e., approximately six times higher). Therefore, a higher BSE surface amount was clearly detected on the LDHc–BSE.

**Figure 4:**
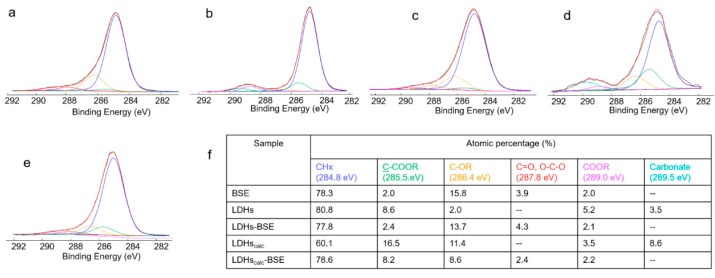
C1s curve fitting of (**a**) BSE, (**b**) LDH, (**c**) LDH–BSE, (**d**) LDHc, and (**e**) LDHc–BSE samples. In panel (**f**), the peak attributions and atomic percentages are reported. Uncertainty in binding energy values was ± 0.2 eV.

For a deeper investigation of the developed hybrid composites, the deconvolution of the high-resolution C1s spectra was performed (see Figure 4).

The BSE C1s spectrum (Figure 4a) clearly evidenced the predominance of C-C or C=C bonds, coded as CHx, as expected in the chemical structure of boswellic acids. However, since this contribution was affected by hydrocarbon contamination, always present in XPS analyses, it cannot be considered as an indication of BSE’s presence on the surface of the hybrid materials. Other peaks, relevant to oxygenated functionalities, seen in most compounds of natural origin, could be observed. Carboxylic acid groups, ascribable to boswellic acids and/or other BSE components, were detected.

In the LDH and LDHc C1s spectra (Figure 4b,d), a carbonate peak was detected at the highest binding energies. Even if the absolute value of the carbonate percentage was higher in the LDHc than in the LDH sample (see Figure 4f), the carbonate’s presence in the calcined clay was significantly lower. Indeed, considering the carbonate/Al2p ratio, it was 1:3 in the LDH and 1:10 in the LDHc sample, evidencing partial carbonate removal, as observed also in the FT-IR and TGA analyses.

Finally, the C1s spectra of both the LDH–BSE and LDHc–BSE samples (Figure 4c,e) showed the absence of carbonate peaks. This finding could be related to the total surface coverage by the plant extract in both composites, rather than to the partial or total replacement of the carbonate by boswellic acids. Indeed, PXRD analysis allowed us to exclude the latter hypothesis. On the other hand, as evidenced by LC-MS analysis (*vide infra*), while methanol extraction completely removed the BSE from the LDH, partial BSE removal was observed for the LDHc–BSE sample, suggesting a strong interaction between the extract compounds and the calcined clay, although intercalation could not be seen.

**Figure 5:**
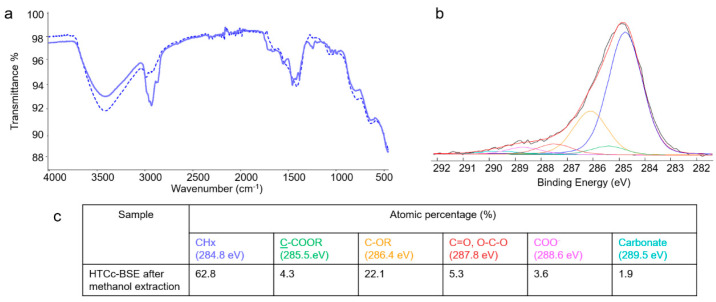
LDHc–BSE (solid line) and LDHc–BSE after methanol extraction (dashed line) FT-IR/ATR spectra (**a**); XPS C1s curve fitting of LDHc–BSE after methanol extraction (**b**), with the relevant peak attributions and atomic percentages (**c**). Uncertainty in binding energy values was ± 0.2 eV.

To shed light on the interaction occurring between the LDHc and the BSE compounds not removed by solvent extraction, a deeper investigation of the LDHc–BSE sample after two methanol extractions was performed. Both the FT-IR and XPS spectra, reported in Figure 5, suggested that the part of the BSE firmly adsorbed on the calcined clay could be ascribed to BAs in their anionic form, which could be electrostatically bound with the positively charged lamellae of the LHDc. Indeed, the C=O stretching of COOH completely disappeared after methanol extraction, as shown by the FT-IR analysis (Figure 3a), while a shift in the COOR peak at lower binding energies (288.6 eV, typical of COO^−^ groups) was observed by XPS, together with the resurfacing of the carbonate contribution already present in the LDHc (see panels b and c).

### 2.4. Targeted RPLC-ESI(−)-FTMS Characterization of Boswellic Acids in BSE and LDH(c)–BSE Hybrid Composites

α-boswellic acid (α-BA) and β-boswellic acid (β-BA), along with their acetylated and oxidized forms, namely 3-acetyl α-boswellic acid (α-ABA), 3-acetyl β-boswellic acid (β-ABA), 11-keto-β-boswellic acid (β-KBA), and 3-acetyl 11-keto-β-boswellic acid (β-AKBA), have been identified as the major bioactive compounds in BSE [33,34,35]. As can be inferred from the chemical structures shown in Figure S1, the acidic properties of boswellic acids (BA) are related to the presence of a common carboxylic acid moiety. Thus, electrospray ionization (ESI) could be recognized as a suitable *soft* ionization approach for the mass spectrometric characterization of these analytes as negative ions ([M − H]^−^). Furthermore, the high resolution/accuracy of the Orbitrap^®^ mass analyzer allowed the use of a very narrow (5 ppm) *m*/*z* extraction window to isolate the contributions of each BA [M − H]^−^ ion from the total ion current chromatogram (TICC). The resulting extracted ion chromatograms (EIC) are shown in Figure S2. Notably, up to nine peaks were observed in the EIC trace pertaining to α-BA and β-BA [M − H]^−^ ions (*m*/*z* 455.3531), thus indicating the presence of multiple isomeric species (see Figure S2A). The unambiguous identification of α-BA and β-BA peaks was based on the comparison of the respective retention times (see Figure S2B) with those obtained for their commercial analytical standards. The mass spectrometric characterization of the remaining isomers will be the subject of future work.

Similarly to what was observed for α-BA and β-BA, the EIC of α-ABA and β-ABA [M − H]^−^ ions (*m*/*z* 497.3636) exhibited more than only two peaks (see Figure S2C). All the HRMS/MS spectra of the ions responsible for these signals were characterized by the presence of a peak at *m*/*z* 59.0138, which is compatible with the detachment of the acetate ion (CH_3_COO^−^) from acetylated ion species. Notably, the signal at *m*/*z* 59.0138 was the only observed peak in the HRMS/MS spectra pertaining to the last two eluting species shown in Figure S2C. As shown in Scheme S1, the formation of the acetate ion as the dominant fragmentation pathway can be rationalized in the case of α-ABA and β-ABA. Here, a β-lactone is formed after the nucleophilic attack of the carboxylate group at its β position, while the acetate ion is released as the leaving group.

Both α-BA and β-BA and their acetylated forms have been detected in the oleogum resin of several *Boswellia* species, including *Boswellia serrata* [36,37]. Schmiech et al. [36] proposed a valuable chromatographic approach for the separation of α-BA, β-BA, α-ABA, and β-ABA. As expected, the acetylation enhanced the retention on a C18 stationary phase, but, more importantly, a similar retention time (RT) shift was observed among the corresponding non-acetylated and acetylated species. Our chromatographic approach was inspired by the work of Schmiech et al. [36] (see Section 3.3.4). Interestingly, a similar RT shift (approximately 3.4 min) was observed for the chromatographic peaks pertaining to α-BA and β-BA and for those that were putatively attributed to α-ABA and β-ABA, seen in Figure S2C.

A single peak was observed in the EIC trace pertaining to β-AKBA (*m*/*z* 511.3429) (see Figure S2E), while an intense peak, followed by a low-intensity signal (labeled as 1d in Figure S2D), was observed in the EIC trace related to β-KBA (*m*/*z* 469.3323). These might be attributed to β-KBA and α-KBA. The most intense and early eluting peak in Figure S2D can be attributed to β-KBA, as it is expected to be dominant in BSE [38]. Moreover, Schmiech et al. reported the higher retention of α-KBA with respect to β-KBA when the two analytes were subjected to RPLC separation on a pentafluorophenyl (PFP) stationary phase [38].

The tentative identification of β-KBA and β-AKBA was also supported by the UV–Vis characterization of BSE. Asteggiano et al. [39] developed an RPLC–UV method for the characterization of several boswellic acids (BA). Here, 250 nm was adopted as the diagnostic absorption wavelength for β-KBA and β-AKBA. Interestingly, perfect alignment was observed between the peaks that were putatively attributed to β-KBA and β-AKBA (see Figure S2D,E) and those that were observed in the RPLC–UV chromatogram recorded at 250 nm (Figure S2F).

In this study, the hyphenation of reversed-phase liquid chromatography and high-resolution mass spectrometry was exploited to quantitatively assess the loading of BAs and their isomers in LDH–BSE and LDHc–BSE hybrid composites. We focused on α-BA, β-BA, and those species that were tentatively identified as α-ABA, β-ABA, β-KBA, and β-AKBA. We also considered all the isomers exhibiting significantly intense peaks in the EIC traces shown in Figure S2A,C. We refer to these species using the following nomenclature, which is related to the labels adopted in Figure S2A,C: BA isomer 3 (peak 3a), BA isomer 4 (peak 4a), BA isomer 5 (peak 5a), BA isomer 6 (peak 6a), BA isomer 7 (peak 7a), ABA isomer 1 (peak 1c), ABA isomer 2 (peak 2c), ABA isomer 3 (peak 3c), and ABA isomer 4 (peak 4c).

**Figure 6:**
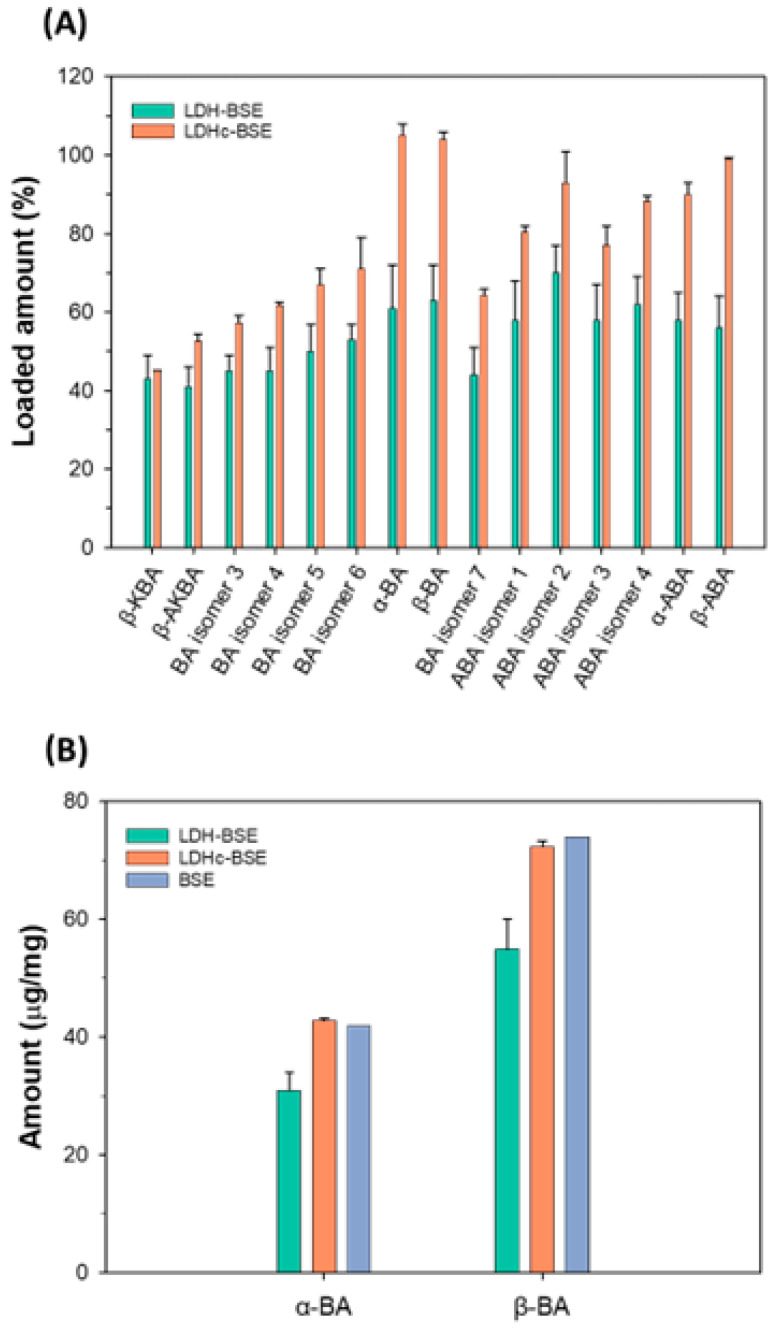
(**A**) Bar charts showing the % loaded amounts of boswellic acids and their isomers in LDH/LDHc–BSE composites. For each analyte, 100% is assumed to correspond to the content embedded in an equal mass of BSE. Here, bar heights are representative of the mean values calculated for three extraction replicates (see Section 3.3.5). The error bars are indicative of the calculated standard deviation. (**B**) Bar charts showing the calculated amounts (μg/mg) of α-BA and β-BA embedded in BSE (blue bars), LDH–BSE (green bars), and calcined LDH–BSE (orange bars). The bar heights are representative of the mean values calculated for three extraction replicates (see Section 3.3.5) in the case of LDH/LDHc composites. The error bars are indicative of the calculated standard deviation.

For each of the analytes of interest, a calibration curve was obtained by plotting the EIC peak area calculated for each of the serial dilutions performed starting from a 100 μg/mL methanolic solution of lyophilized BSE (see Section 3.3.6). The mathematical equations of the calibration curves are shown in Table S1. As stated in Section 3.3.6, the outcome of the interpolation of the normalized peak area response with the calibration curve was interpreted as the percentage amount of the loaded analyte into a given mass of the LDH/LDHc–BSE composite, with respect to the content of such analyte in the same mass of BSE. The results are shown in Table S2A and graphically summarized in Figure 6A.

The loaded amount of the analytes of interest was found to be remarkably higher in the LDHc–BSE than in the LDH–BSE composites, except for the early eluting (i.e., more polar) species, namely β-KBA and β-AKBA. This is consistent with the improved BSE loading capability of the LDHc system that was assessed by the thermal analysis (see Section 2.1).

Notably, the highest loading discrepancies between the two systems were observed for α-BA, β-BA, and their acetylated forms (α-ABA, β-ABA). To further support these findings, we quantified the absolute amounts of α-BA and β-BA (see Section 3.3.6) in the inorganic–organic composites and compared the outcomes with the estimated amount in BSE. The results are shown in Table S2B and graphically summarized in Figure 6B. The mathematical equations of the calibration curves are shown in Table S1B. Consistently with what is shown in Figure 6A, the estimated amount of α-BA and β-BA in the LDHc–BSE system was comparable with the one observed for pure BSE. Conversely, a lower amount was observed in LDH–BSE composites.

Besides the loading of BA into the LDH–BSE and LDHc–BSE systems, the release of BAs in aqueous media needed to be assessed for a more thorough characterization of the two composites. For such a purpose, phosphate-buffered saline solution (PBS) was chosen as the release medium. Indeed, PBS represents a simple body fluid model that can mimic the biological environment in which the released BAs are expected to exhibit their bioactivity. As described in Section 3.3.7, the composites were dispersed into the PBS solution to a nominal concentration of 100 μg/mL and kept under gentle stirring for 2 h at a physiological body temperature (37 °C). Thereafter, the released BAs were extracted with chloroform and subjected to RPLC–ESI(−)–FTMS analysis. The contribution of each BA to the release profile (RP) was calculated as the percent ratio between the observed instrumental response (EIC peak area) and the sum of the responses calculated for all of the species of interest (i.e., β-KBA, β-AKBA, BA isomers 1, 2, 3, α-BA, β-BA, α-ABA, and β-ABA). Notably, the released amount of ABA isomers 1, 2, and 3 was not sufficiently high to ensure reliable EIC peak integration. Hence, the contribution of the latter species to the RP was neglected. Figure S3 offers a schematic representation of the calculated RP for both LDH–BSE and LDHc–BSE composites. In addition, Figure S3 shows a comparison of the two RPs with the BA profile calculated for pure BSE. The latter was determined after the RPLC–ESI(−)–FTMS analysis of a 1 μg/mL methanol solution of pure BSE, corresponding to calibration level 2 (see Section 3.3.6). Indeed, the EIC peak area values calculated for BAs in level 2 exhibited the same order of magnitude as the EIC areas calculated for BAs released in PBS from the two composites.

The RPs of both the LDH–BSE and LDHc–BSE systems showed similar discrepancies with respect to the BA profile of pure BSE. In both cases, the dissimilarities were ascribable to the higher tendency of KBA to be released in the PBS medium. This is consistent with the lower hydrophobicity of KBA with respect to the other BAs enclosed in pure BSE, which could be easily inferred from the elution order observed on the C18 stationary phase (see Figure S2). However, as with all BAs, KBA is slightly soluble in aqueous media. Indeed, the released amount of KBA in PBS from the LDH–BSE and LDHc–BSE systems was, respectively, estimated as (1.51 ± 0.03)% and (1.12 ± 0.02)% of the KBA content in 100 μg of pure BSE. These data (mean ± standard deviation) refer to triplicates of the release assay described in Section 3.3.7.

### 2.5. Morphological Characterization of LDH(c)–BSE Hybrid Composites by Scanning Electron Microscopy

**Figure 7:**
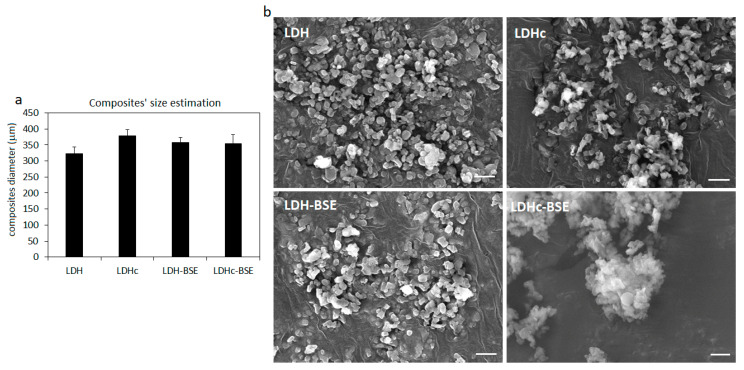
Composites’ morphology and size in SEM images. (**a**) The calcination procedure (LDHc) as well as the BSE loading (LDH–BSE) determined a slight increase in the LDH size. (**b**) SEM images reported a similar round-like shape and morphology for all the composites. Image magnification = 10,000×, bar scale = 1 μm.

The morphological analysis of the obtained composites, as well as their size in terms of diameter range, was provided by SEM imaging (some representative images of the diameter calculation can be observed in Supplementary Figure S4). Results are reported in Figure 7. The SEM images (Figure 7b) revealed a similar round-shaped morphology for all of the composites; size analysis (Figure 7a) reported an average size of 322 (±21) μm for the LDH composites, which was slightly increased when the calcination process was applied (LDHc, average size of 379 ± 18 μm) and the BSE was loaded (LDH–BSE, average size 358 ± 15 μm). Conversely, the average size of the calcinated composites (LDHc) was not increased after the BSE loading (LDHc–BSE, average size 354 ± 27 μm).

### 2.6. Antimicrobial Activity of BSE-Based Composites

The increasing ability of bacteria to develop defense mechanisms against the biological activity of the conventional antibiotics highlights the urgent need for alternative active principles able to counteract pathogen infections. Natural compounds and their extracts are known to possess intrinsic antibacterial activity, and they normally present minimal side effects due to their natural origins. Among the large class of such natural extracts, *Boswellia serrata* (BSE) represents an interesting source of antibacterial active compounds. It has been previously reported in the literature that this extract is largely composed of terpene substances that are known to be able to bind to the membrane, thus causing the formation of irreversible pores, and they can lower protein synthesis, reducing ATP consumption and interfering with the quorum-sensing signaling during biofilm formation [40].

Beyond these promising intrinsic antibacterial properties, it is also well known that the bioactivity of natural compounds and their extracts is limited by their chemical instability and poor aqueous solubility, which severely limit their effectiveness [41]. To overcome this limitation, the composites here developed were intended to stabilize the bioactivity of the BSE extract in solution, to improve its efficacy when a bacterial infection is ongoing, as a potential alternative to conventional antibiotics. In particular, the major aim of the developed composites was to increase the bioavailability of BSE in such a liquid environment in order to better exploit its inherent antibacterial properties thanks to the carrier role of the LDH or LDHc. Accordingly, the composites were placed in direct contact for 24 h with viable colonies of *Escherichia coli* (*E. coli, Gram−*)*, Pseudomonas aeruginosa* (*P. aeruginosa, Gram−*)*, Staphylococcus aureus* (*S. aureus, Gram+*)*,* and *Staphylococcus epidermidis* (*S. epidermidis, Gram+*) cultivated in their liquid broth media. Bacteria were cultivated at a high density (1x10^5^ bacteria/mL) to resemble a pathological scenario and the results of the composites (LDH–BSE and LDHc–BSE) were compared to those of the bare extract (BSE) to verify whether they introduced an improvement in terms of antibacterial activity.

Results are reported in Table 2. In general, the LDH–BSE reported the lowest number of viable bacteria for all the tested strains in comparison to the bare extract (BSE). In particular, the results were statistically significant (*p* < 0.05, indicated by § in the Table 2) for *E. coli* and *S. aureus,* where the reduction in the number of viable colonies was 63% and 34%, respectively. For other strains (*P. aeruginosa* and *S. epidermidis*), a reduction of 20% and 26% was observed, but the results were not statistically significant (*p* > 0.05). Differently, LDHc–BSE showed significant results in comparison to the bare extract (BSE) only for *E. coli* (*p* < 0.05, indicated by §), whereas a non-significant reduction in viable colonies was reported for *P. aeruginosa*, *S. aureus*, and *S. epidermidis*. However, in terms of bioactivity, the difference observed between the LDH–BSE and LDHc–BSE could be ascribed to the stronger interaction between the extract and the calcined clay, as previously observed in the LC-MS results. Finally, as might be expected considering that the antibacterial active ingredient was related to the boswellic acids present in the extract, the composites not loaded with the extract (LDH and LDHc) showed no inhibitory activity compared to the extract per se.

To compare the obtained results, only a very limited body of literature can be found. In particular, Bi et al. reported similar findings showing the possibility to enhance BSE’s bioactivity by loading the extract into hydroxyapatite carboxymethyl cellulose composites (named HAP/BE/CMC) [42]. The antibacterial assay results demonstrated that the HAP/BE/CMC composites were able to significantly reduce the propagation of *Bacillus cereus* and *Pseudomonas aeruginosa* via the agar diffusion test in comparison to the unloaded hydroxyapatite. In the context of food packaging, Narasagoudr et al. demonstrated that the incorporation of BSE into chitosan poly(vinyl alcohol) films was significantly more effective than BSE alone in reducing the infection of *Escherichia coli, Staphylococcus aureus,* and *Candida albicans* [43] in the agar diffusion assay. Therefore, our results seem to be in line with the previous literature suggesting the higher bioactivity of BSE loaded into delivery systems aimed at improving its bioavailability, even if the exploitation of different methodologies (CFU count and agar diffusion test) does not allow us to properly compare the overall efficacy of the BSE-based systems. However, according to our findings, it can be speculated that, in particular, the composite LDH–BSE represents a promising candidate for further investigation due to its higher bioactivity towards both Gram − and Gram + strains in comparison to the bare extract.

### 2.7. Antinflammatory Activity of BSE-Based Composites

To further verify the superior bioactivity of the composites in comparison to the extract, the anti-inflammatory properties were tested. Boswellic acids have been largely reported in the literature to be able to interfere with many pathways involved in the inflammatory cascade, such as 5-lipoxygenase, human leukocyte elastase, and toposiomerase I and II, as well as Ca^2+^ and MAPK signaling in various blood cells [44]. A specific role of boswellic acids has been also clarified regarding their antioxidant properties, as they are involved in the inhibition of the pro-inflammatory NF-κB activator and in the modulation of other relevant inflammatory molecular targets, such as AP-1 and β-catenin, as well as enzymes like COX-2 and pro-inflammatory cytokines (TNF-α, IL-1β) [45]. Accordingly, a pro-inflammatory condition was induced by oxidative stress chemically generated by hydrogen peroxide addition into the culture medium; afterwards, supernatants were collected and used to cultivate cells, as previously reported by the authors [46,47,48]. In such a pro-inflammatory scenario, the well-known scavenger ability of the boswellic acids [49,50] was expected to significantly reduce the amount of the cytotoxic oxygen- and nitrogen-derived (ROS) active species, thus protecting cells from intracellular accumulation.

**Figure 8:**
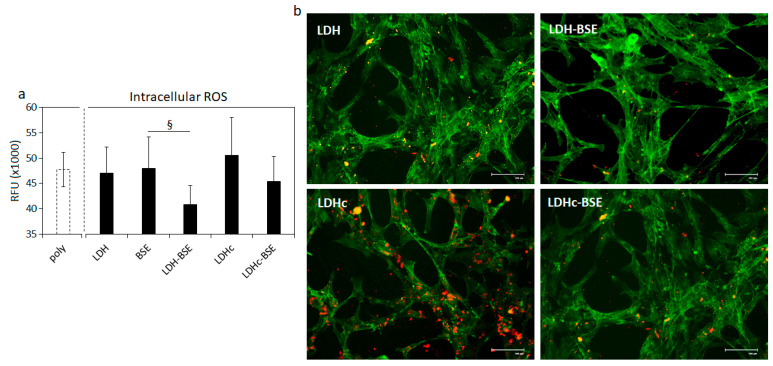
ROS intracellular accumulation. (**a**) The quantification of the RFU due to ROS intracellular accumulation revealed that the LDH–BSE was significantly more efficient in comparison to BSE in reducing the internalization of toxic species (*p* < 0.05, indicated by §); (**b**) in general, fluorescent images demonstrated that the BSE delivered by the composites reduced the amount of internalized toxic species, suggesting improved bioactivity. Bars represent means ± dev.st of six replicates. Image bar scale = 100 μm; ROS are stained in red; cells’ cytoskeletons are stained in green.

Results are reported in Figure 8. The first interesting outcome is related to the comparable RFU values reported for the controls (cells cultivated in polystyrene—POLY) and BSE, shown in Figure 8a (not significant, *p* > 0.05); such a result confirms the poor bioactivity of the extract per se when applied in a liquid environment due to the poor solubility, which prevented the scavenger activity towards the generated ROS. Similarly, the unloaded clays (i.e., LDH and LDHc) did not display any efficacy in reducing the amount of free ROS (Figure 8a, *p* > 0.05) in comparison with controls. Conversely, the LDH–BSE and LDHc–BSE composites determined a reduction in the intracellular amount of ROS, thus demonstrating the bioactivity of the BSE delivered by the hydrotalcite carrier; fluorescent images (Figure 8b) clearly demonstrated the difference in terms of the intracellular ROS amount (stained in red) between the unloaded (LDH and LDHc) and BSE-loaded (LDH–BSE and LDHc–BSE) systems. In particular, the LDH–BSE showed a significant reduction in ROS in comparison to BSE (Figure 8a, *p* < 0.05 indicated by §), thus demonstrating the ability of the composite to increase the bioactivity of BSE. However, the results were not significant when the LDHc–BSE was compared to BSE (*p* > 0.05), thus confirming the observations made above for the antibacterial assay in relation to the lower ability of these composites to deliver the loaded BSE in comparison to the LDH–BSE ones. These results are in line with the work published by Mehta et al. [7]; by loading boswellic acid into a proniosomal gel for topic application, they observed a significant reduction in paw edema in rats induced by the pharmacological administration of pro-inflammatory carrageenan. The observed effect was correlated by the authors to the ability of the BSE to reduce in situ the cells’ adsorption of toxic compounds due to oxidative stress, thus strongly reducing the activation of the pro-inflammatory cascade.

Therefore, in accordance with the antibacterial assay results, it can be speculated that the LDH–BSE composite represents a promising system for the delivery and enhancement of BSE’s bioactivity.

## 3. Materials and Methods

### 3.1. Materials

Synthetic hydrotalcite (magnesium aluminum hydroxycarbonate, CAS 11097-59-9, molecular weight 603.98 g/mol), coded as LDH, was purchased from Sigma-Aldrich (Merck, Milan, Italy). *Boswellia serrata Roxb* powder extract (containing 65% boswellic acids, CAS 97952-72-2), coded as BSE, was purchased from Farmalabor S.p.A. (Apulia, Italy). All solvents and reagents were purchased from Sigma-Aldrich (Merck, Milan, Italy), unless otherwise specified.

### 3.2. Preparation of the Hybrid Composites Based on LDH and Calcined LDH

The loading of BSE was achieved by equilibrating LDH with a 0.05 M water/ethanol (25/75 *v*/*v*) solution, (considering a theoretical milliequivalent ratio between organic anions and CO_3_^2−^ of 2.5:1) for 24 h at room temperature. The recovered solid, obtained by filtration, was washed three times with distilled water and then dried at 60 °C until a constant weight was reached. Notably, it was expected that minimal or no anion exchange would occur for the LDH in its “as-received” carbonate form, since carbonate is preferentially sorbed in the material, thus preventing further significant anion exchange. In this respect, only a surface adsorption process of BSE was expected to occur. On the other hand, carbonate can be removed by thermal decomposition, leading to carbon dioxide, by heating the hydrotalcite at temperatures higher than 400 °C, as reported by Miyata [51]. Therefore, a calcination process was performed on the LDH, by heating it at 450 °C for 2 h [52]. The recovered solid (coded as LDHc) was then loaded using the same amount of BSE used for the non-calcined clay but replacing water with CO_2_-free deionized water. Both LDH–BSE and LDHc–BSE samples were stored in a desiccator until use.

### 3.3. Physical–Chemical Characterization of the Hybrid Composites

#### 3.3.1. Thermogravimetric Analysis (TGA)

The thermal behavior of the hybrid composites was assessed by a PerkinElmer TGA-400 instrument (Perkin Elmer, Milan, Italy), heating 5–10 mg of the samples between 30 and 600 °C. The analyses were performed in nitrogen, with a gas flow set at 20 °C/min. Data were recorded by means of the TGA Pyris software (version 13.3.1.0014).

#### 3.3.2. Powder X-ray Diffraction (PXRD) Measurements

For powder X-ray diffraction (PXRD) data analysis, the patterns were collected using a Rigaku Rint2500 laboratory diffractometer with a rotating Cu anode. The instrument operated at 50 kV and 200 mA in Debye–Scherrer geometry. The diffractometer was equipped with an asymmetric Johansson Ge (111) crystal to select the monochromatic CuKα_1_ radiation (λ = 1.54056 Å), along a silicon strip Rigaku D/teX Ultra detector. The data were collected in the range of 5 to 100° (2ϑ) with a step size of 0.02° (2ϑ) and a counting time of 6 s/step. To perform the measurements, each powder sample was introduced into a glass capillary with a diameter of 0.5 mm and mounted on the goniometer’s axis. The capillary was rotated during the measurement to enhance the randomization of the individual crystallites’ orientations and minimize the potential impact of the preferred orientation.

#### 3.3.3. Bulk and Surface Chemical Characterization

FT-IR/ATR analyses were conducted using a Spectrum Two-PE instrument endowed with a universal ATR accessory (UATR, Single Reflection Diamond/ZnSe) supplied by PerkinElmer. For each of the relevant samples, FT-IR/ATR spectra were recorded from 400 to 4000 cm^−1^ with a 4 cm^−1^ resolution.

XPS analyses were performed using a scanning microprobe, PHI 5000 VersaProbe II, purchased from Physical Electronics (Chanhassen, MN, USA). The instrument was equipped with a micro-focused monochromatized AlKα X-ray radiation source. The samples were examined in HP mode with an X-ray take-off angle of 45° (instrument base pressure ~10^−9^ mbar). The size of the scanned area was approximately 1400 μm × 200 μm. Wide scans and high-resolution spectra were recorded in FAT mode for each sample, setting pass-energy values equal to 117.4 eV and 29.35 eV, respectively. To fit the high-resolution spectra, the commercial MultiPak software version 9.9.0.8 was used. Adventitious carbon C1s were set as the reference charge (284.8 eV).

#### 3.3.4. RPLC–ESI–FTMS Instrumentation and Operating Conditions

The RPLC–ESI(−)–FTMS analysis was performed using an LC-MS platform implementing an Ultimate 3000 HPLC quaternary chromatographic system and a Q Exactive high-resolution quadrupole–Orbitrap mass spectrometer (Thermo Fisher, West Palm Beach, CA, USA). Here, the chromatographic column effluent was transferred to the heated electrospray ionization (HESI) interface (Thermo Fisher, West Palm Beach, CA, USA) mounted on the mass spectrometer. The RPLC separations of BAs and their isomers were performed using a C18 Ascentis Express column (15 cm length, 2.1 mm internal diameter) packed with core–shell 2.7 μm particles (Supelco, Bellefonte, PA, USA) and operated at a 0.2 mL/min flow rate. A 5 μL sample volume of the resulting solution was subjected to RPLC–ESI(−)–FTMS/MS analysis.

The separation of BAs and their isomers was obtained using a modified version of the chromatographic approach proposed by Schmiech et al. [36]. The following multi-step binary elution gradient, based on a 80:20 *v*/*v* methanol/water mixture as phase A and methanol as phase B, both containing a 2.5 mM nominal concentration of ammonium acetate and ammonia, was adopted:

- 0–2 min—isocratic at 10% B;
- 2–13 min—linear increase in B from 10% to 20%;
- 13–20 min—linear increase in B from 20% to 70%;
- 20–27 min—isocratic at 70% B;
- 27–29 min—linear decrease in B from 70% to 10%;
- 29–40 min—isocratic re-equilibration at 10% B.

The parameters of the HESI interface and of the ion optics of the Q-Exactive spectrometer were set as follows:

- sheath gas flow rate: 40 a.u.;
- auxiliary gas flow rate: 15 a.u.;
- spray voltage: −3 kV;
- capillary temperature: 200 °C;
- S-lens RF level: 60.

The mass spectrometer was operated at its maximum resolving power (140,000 at *m*/*z* 200) for both full-scan MS and MS/MS experiments. Full-scan high-resolution MS spectra were acquired in a 300–700 *m*/*z* interval. Here, the Automatic Gain Control (AGC) level was set to 1 × 10^6^, with a maximum injection time of 100 ms. Normalized collision energy (NCE) of 60 units was adopted for MS/MS experiments, while the AGC level was set to 2 × 10^5^, with a maximum injection time of 100 ms. The spectrometer was calibrated daily by infusing, at a 5 μL/min flow rate, calibration solutions provided by the instrument manufacturer for negative polarity acquisitions. As a result, mass accuracy always better than 5 ppm was achieved.

#### 3.3.5. Extraction of BSE from LDH–BSE and LDHc–BSE Composites

The lyophilized BSE exhibited excellent solubility in methanol (MeOH). Hence, this solvent was initially selected as the extraction medium for the recovery of BSE in the LDH–BSE and LDHc–BSE composites. For such a purpose, the inorganic–organic composite was dispersed in pure MeOH to a nominal concentration of 1 mg/mL. The mixture was vigorously stirred (1 min) using a vortex mixer and the extraction of the organic material was further supported by a further sonication step for 10 min. A DU-32 ultrasonic bath (Argo Lab, Carpi, Italy), operating at 40 KHz frequency, 120 W power, and 25 °C temperature, was adopted for the latter purpose. Thenceforth, the separation between the solid phase and the extraction medium was obtained after centrifugation (4500× *g*) for 10 min. The supernatant was withdrawn and diluted by a 1:10 factor in pure methanol to an overall volume of 1 mL. The sample was spiked with 50 μL of a 52.5 μg/mL methanolic solution of oleic acid (internal standard) prior to the RPLC–ESI(−)–FTMS analysis.

As proven by the FT-IR/ATR analysis of the composites before and after the extraction step (see Section 2.3), this extraction procedure was efficient in determining the quantitative recovery of BSE from LDH–BSE composites. Conversely, the BSE content was only partially extracted in the case of LDHc–BSE systems. To increase the extraction yield for the latter composites, we decided to adopt an alternative extraction procedure based on the acidic pretreatment of the matrix. Indeed, the LDHc was proven to be quantitatively solubilized in an aqueous hydrochloric acid (HCl) solution at pH 1.2. Hence, we dispersed the LDHc–BSE in the same acidic aqueous medium to a nominal concentration of 1 mg/mL. The heterogeneous mixture was vigorously stirred for 1 min using a vortex mixer. Thereafter, pure chloroform was added in a 1:1 volume ratio with respect to the acidic aqueous phase. The mixture was stirred again for 1 min and clear phase separation was obtained after centrifugation (4500× *g*) for 10 min. Then, 1 mL of the organic phase was recovered and subjected to solvent evaporation under nitrogen flow. The dry residue was redissolved in 1 mL of pure MeOH. The resulting mixture was diluted by a 1:10 factor in pure methanol to an overall volume of 1 mL. Moreover, in this case, the sample was spiked with 50 μL of a 52.5 μg/mL methanolic solution of oleic acid (internal standard) prior to the RPLC–ESI(−)–FTMS analysis.

#### 3.3.6. Quantification of Boswellic Acids in LDH–BSE and LDHc–BSE Composites

Two analytical strategies were adopted to assess the extent of the loading of BAs in LDH–BSE and LDHc–BSE composites.

The first was based on the construction of calibration curves for α-BA, β-BA, α-ABA, β-ABA, and their isomers, starting from consecutive dilutions of a 1 mg/mL methanolic solution of lyophilized BSE. Specifically, 1 mL of the following calibration levels was obtained: level 1 (0.1 μg/mL), level 2 (1 μg/mL), level 3 (5 μg/mL), level 4 (10 μg/mL), level 5 (50 μg/mL), and level 6 (100 μg/mL). Each of the calibration solutions was spiked with 50 μL of a 52.5 μg/mL methanolic solution of oleic acid (ISTD) prior to the RPLC–ESI(−)–FTMS analysis. As shown in Section 3.3.5, the extraction procedure involved the dispersion of the composites in the extraction medium to a nominal concentration of 1 mg/mL. At the end of the process, the extracts were diluted by a 1:10 factor. Hence, the BA content of the diluted extract could be considered representative of 100 μg of organic–inorganic composite. On the other hand, the BA amount in the upper calibration level (level 6) was representative of the BA content in the same mass of BSE. Therefore, for each of the analytes of interest, the result of the interpolation of the analytical response (ISTD-normalized EIC areas) with the calibration curve could be interpreted as follows: it represented the loaded amount of a BA in a given mass of the composite, expressed as the percentage of the content of this BA in the same mass of BSE.

The second quantitative approach aimed at the determination of the absolute amounts of α-BA and β-BA in BSE as well as in the composites. For such a purpose, an external calibration approach was adopted. Five calibration levels, corresponding to equimolar mixtures of α-BA and β-BA, were prepared: level 1 (0.1 μg/mL), level 2 (1 μg/mL), level 3 (5 μg/mL), level 4 (10 μg/mL), level 5 (50 μg/mL). First, 1 mL of each of the calibration levels was spiked with 50 μL of a 52.5 μg/mL methanolic solution of oleic acid (ISTD) prior to the RPLC–ESI(−)–FTMS analysis. The calibration curves were exploited to determine the absolute amounts of α-BA and β-BA in a 100 μg/mL methanolic solution of a BSE, as well as in the BSE extracts obtained from LDH/LDHc–BSE composites (see Section 3.3.5).

#### 3.3.7. Release of Boswellic Acids from LDH(c)–BSE in Aqueous Environment

The release profile of BAs was evaluated in a phosphate-buffered saline solution (pH 7.4). For such a purpose, the LDH–BSE and LDHc–BSE composites were dispersed into the release medium to a nominal concentration of 100 μg/mL. The heterogeneous mixture was gently stirred for 2 h at 37 °C. Thereafter, a 2 mL aliquot was withdrawn and filtered using 0.45 μm PTFE filters. Then, 2 mL of chloroform was added to the resulting liquid phase and the system was vigorously stirred using a vortex mixer. Next, 1 mL of the organic phase was recovered and subjected to solvent evaporation under controlled nitrogen flux. The dry residue was redissolved in pure methanol and subjected to RPLC–ESI–MS analysis.

#### 3.3.8. Morphology Evaluation

Composites’ morphologies before and after BSE loading were visually evaluated by scanning electron microscopy (SEM, JEOL JSM-IT500, from JEOL, Milan, Italy); briefly, composites were dehydrated by the alcohol scale (70–90–100%, 20 min each) and fixed onto SEM stubs by conductive glue prior to being visualized at various magnifications. Moreover, the size of the single composite was estimated by the SEM software (https://www.jeol.com/products/scientific/sem/, SMILE VIEW™ Lab, from JEOL, Milan, Italy) in terms of the diameter range by reporting the average size (± dev.st) of 20 single nanoparticles of 3 replicates (60/composite in total).

### 3.4. Antibacterial Evaluation

The antibacterial activity of the BSE, LDH, LDH–BSE, LDHc, and LDHc–BSE systems against two Gram + (*Staphyloccocus aureus* ATCC 43300 and *Staphylococcus epidermidis* ATCC 51625; American Type Culture Collection—ATCC) and two Gram − pathogenic bacterial strains (*Escherichia coli* ATCC 25922 and *Pseudomonas aeruginosa* ATCC 15422) was evaluated by direct contact between the bacterial strains and the composites. These commercial strains, in particular, were selected at this stage of the composites’ validation due to the manufacturer’s indications of these as reference strains for drug discovery and innovative antibacterial compound studies; moreover, both *S. aureus* and *S. epidermidis* are certified as drug-resistant, thus allowing for the study of BSE as a potential alternative to conventional antibiotics. According to Dianhai et al., [42] reporting the concentration of 100 μg/mL as the minimum inhibitory concentration (MIC) of BSE, 1 mL of bacterial suspension at a concentration of 1 × 10^5^ CFU/mL was directly exposed to the above-mentioned amount of the composite. Then, specimens were incubated in an incubator with controlled humidity and temperature (95% and 37 °C, respectively) for 24 h. Afterwards, the number of viable bacteria was evaluated by the colony forming unit (CFU) count as previously detailed by the authors [53,54].

### 3.5. Anti-Inflammatory Efficacy

The composites’ ability to act as antioxidants via scavenging activity was evaluated regarding their ability to protect cells from the intracellular accumulation of toxic active species in a pro-inflammatory scenario. Accordingly, oxidative stress was chemically induced by adding hydrogen peroxide (H_2_O_2_, 3 h/day, 300 μM) into the medium to generate oxygen-derived toxic active species (ROS), as previously shown by the authors [46,47,48]. Accordingly, H_2_O_2_ was added to the medium in the presence of the composites and agitated (100 rpm) at room temperature for 3 days. Afterwards, 1 mL of this solution was added to a new 24-multi-well plate containing human bone marrow mesenchymal stem cells (hBMSC, 2x10^4^ cells/well, from PromoCell C-12974) and incubated at 37 °C for 24 h. To verify intracellular ROS internalization, the specific CellRox reagent (CellROX ™ Deep Red Reagent Kit, from Thermo Fisher Scientific, Milan, Italy) was applied and cells were further co-stained with phalloidin (Alexa Fluor 488 Phalloidin, from Thermo Fisher Scientific, Milan, Italy) to visualize cytoskeleton F-actin filaments. Images were collected by a confocal microscope (Leica TCS SP8 LIGHTNING confocal laser scanning microscope). Finally, to quantify the ROS amount, the CellRox fluorescent signals (expressed as relative fluorescent units—RFU) were detected at 640 nm and 665 nm excitation and emission wavelengths, respectively. Cells cultivated in a regular medium with H_2_O_2_ were considered as controls to compare the results from specimens.

### 3.6. Statistical Data Analysis

Experiments were performed using six replicates for each assay. Results were statistically analyzed using the SPSS software (v.20.0, IBM, Armonk, NY, USA). First, data’s normal distribution and homogeneity of variance were confirmed by the Shapiro–Wilk and the Levene test, respectively; then, groups were compared by one-way ANOVA using Tukey’s test as a post-hoc analysis. Significant differences were established at *p* < 0.05.

## 4. Conclusions

A lamellar solid layered double hydroxide (LDH), corresponding to a synthetic magnesium aluminum carbonate, was evaluated as such or after calcination (LDHc) for its potential as a carrier of the bioactive compounds included in *Boswellia serrata* extract, poorly soluble in water. Data obtained by TGA, PXRD, FT-IR/ATR, and XPS consistently suggested the disruption of the original LDH structure upon calcination. An increase in the amount of BSE-related compounds, especially α- and β-boswellic acids and the corresponding acetylated derivatives, embedded into the inorganic material was also evidenced by LC-MS analyses when the LDHc was adopted. On the other hand, in vitro tests of the antibacterial and anti-inflammatory activity exerted by the LDH(c)–BSE composites indicated better performance when the LDH was adopted, with significant improvements with respect to BSE as such, with particular reference to the anti-inflammatory properties. These results suggest that anionic forms of the compounds occurring in BSE were more retained in the LDHc, likely due to their roles as substituents of the carbonate anions originally included in the material structure, as also confirmed by FT-IR and XPS. This feature limited their subsequent release in the aqueous environment adopted for tests of biological activity. Based on these outcomes, this study opens interesting perspectives for the development of composites including LDHs as deliverers of BSE’s bioactive compounds, exhibiting interesting combinations of chemical, physical, and biological properties when compared to those of the bare extract.

## Figures and Tables

**Table 1:** Surface composition by XPS analysis of BSE, hydrotalcites, and BSE-embedded hydrotalcites.

Sample	Atomic %						
	C1s	O1s	Ca2p	Na1s	Al2p	Mg1s	Si2p
BSE	86.1	12.6	0.9	0.4	--	--	--
LDH	47.4	42.3	--	--	5.2	3.7	1.5
LDH–BSE	76.4	19,1	--	--	1.5	1.0	2.1
LDHc	16.8	55.2	--	--	11.2	16.8	0.7
LDHc–BSE	79.1	16.7	--	--	1.6	1.3	1.2

**Table 2:** Colony forming unit (CFU) counts of viable bacteria after being in direct contact with composites for 24 h. Results are reported as means ± dev.st ×10^7^; § = *p* < 0.05 vs. BSE. Experiments were performed using six replicates.

	*E. coli*	*P. aeruginosa*	*S. aureus*	*S. epidermidis*
BSE	124 (±5)	15 (±3)	18 (±3)	15 (±10)
LDH	150 (±1)	15 (±4)	20 (±1)	18 (±3)
LDH–BSE	46 (±2) ^§^	12 (±3)	12 (±3) ^§^	11 (±1)
LDHc	120 (±1)	16 (±8)	17 (±6)	29 (±3)
LDHc–BSE	68 (±2) ^§^	12 (±1)	15 (±1)	20 (±3)

## Data Availability

The data presented in this study are available herein.

## Supplementary Materials

The following supporting information can be downloaded at: https://www.mdpi.com/article/10.3390/molecules28186449/s1, Scheme S1: Putative fragmentation mechanism illustrating the formation of the acetate ion (*m*/*z* 59.0139) from precursor ions exhibiting the same structural features of the first of the five condensed rings constituting the molecular backbone of acetyl α-boswellic acid (α-ABA) and 3-acetyl β-boswellic acid (β-ABA); Table S1: (A) Parameters of the calibration curves obtained after serial dilutions and analysis of a methanolic solution of lyophilized BSE (see Section 3.3.6 for details). (B) Parameters of the calibration curves for the absolute quantification of α-BA and β-BA (see Section 3.3.6 for details). Table S2: (A) Estimated percent loaded amount of boswellic acids in the LDH-BSE and LDHc-BSE composites. (B) Estimated loaded amount (μg/mg) of α-BA and β-BA in BSE, LDH-BSE and LDHc-BSE composites. Figure S1: Chemical structures of α-boswellic acid (α-BA), β-boswellic acid (β-BA), 11-keto-β-boswellic acid (β-KBA), 3-acetyl α-boswellic acid (α-ABA), 3-acetyl β-boswellic acid (β-ABA), and 3-acetyl 11-keto-β-boswellic acid (β-AKBA). Figure S2: Extracted ion chromatograms (EIC) referring to the RPLC-ESI(−)-FTMS analysis of a methanolic solution (100 μg/mL) of lyophilized BSE. The EIC traces were obtained by setting a 5 ppm extraction window centred on the theoretical *m*/*z* of the [M − H]^−^ ions of (A) α-BA and β-BA (*m*/*z* 455.3531), (C) α-ABA, and β-ABA (497.3636), (D) β-KBA (469.3323), and (E) β-AKBA (*m*/*z* 511.3429). Panel (B) displays the overlap of two EIC traces referring to the RPLC-ESI(−)-FTMS analysis performed on each of the two equally concentrated (10 μg/mL) methanolic solutions of the α-BA and β-BA analytical standards. Panel (F) shows the RPLC-UV-DAD chromatogram recorded at 250 nm for a 1 mg/mL methanolic solution of lyophilized BSE. Figure S3: Ring charts displaying the calculated BA profile in pure BSE and the profile of BA released in PBS solution from LDH-BSE and LDHc-BSE composites. Figure S4: SEM visual analysis of the developed composites’ morphology. Images are representative for the diameter size estimation of the single nanocomposites randomly selected; calculation was obtained by the SEM software. Images magnification = 8000×, bar scale = 2 µm.

## Sample Availability

Samples of the natural extracts, standard compounds and composites are available from the authors.
