# Supplementary material for: Development, Analytical Characterization, and Bioactivity Evaluation of Boswellia serrata Extract-Layered Double Hydroxide Hybrid Composites
Source: Molecules. 2023 Sep 5;28(18):6449. doi: 10.3390/molecules28186449 (PMC10537998; doi:10.3390/molecules28186449)
Supplement: Supplementary file 1 [file molecules-28-06449-s001.zip › molecules-2540539-supplementary.pdf]

Supporting Information

Development, Analytical Characterization, and Bioactivity Evaluation of *Boswellia serrata* Extract-Layered Double Hydroxide Hybrid Composites

Stefania Cometa [1], Francesco Busto [2,3], Andrea Castellaneta [2], Andrea Cochis [4], Ziba Najmi [4], Rosanna Rizzi [5], Ilario Losito [2,6] and Elvira De Giglio [2,3,6,*]

[1] Jaber Innovation s.r.l., Via Calcutta 8, 00144 Rome, Italy; stefania.cometa@jaber.it
[2] Department of Chemistry, University of Bari, Via Orabona 4, 70126 Bari, Italy; f.busto3@studenti.uniba.it (F.B.); andrea.castellaneta@uniba.it (A.C.); ilario.losito@uniba.it (I.L.)
[3] INSTM, National Interuniversity Consortium of Materials Science and Technology, Via G. Giusti 9, 50121 Florence, Italy
[4] Center for Translational Research on Autoimmune and Allergic Disease, CAAD, Department of Health Sciences, Università del Piemonte Orientale UPO, 28100 Novara, Italy; andrea.cochis@med.uniupo.it (A.C.); ziba.najmi@uniupo.it (Z.N.)
[5] Institute of Crystallography, National Research Council (CNR), Via G. Amendola, 122/o, 70126 Bari, Italy; rosanna.rizzi@ic.cnr.it
[6] SMART Inter-Department Research Center, University of Bari, Via Orabona 4, 70126 Bari, Italy
* Correspondence: elvira.degiglio@uniba.it

Supplementary schemes

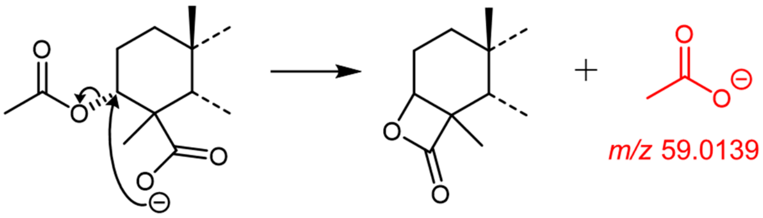

Scheme S1.

Putative fragmentation mechanism illustrating the formation of the acetate ion (*m/z* 59.0139) from precursor ions exhibiting the same structural features of the first of the five condensed rings constituting the molecular backbone of acetyl α-boswellic acid (α-ABA) and 3-acetyl β-boswellic acid (β-ABA).

Supplementary tables

Table S1.

A) Parameters (mathematical equation and coefficient of determination) of the calibration curves obtained after serial dilutions and analysis of a methanolic solution of lyophilized BSE (see **Section 3.3.6** for details). Here "y" represents the analytical response, *i.e.*, the EIC peak area normalized to the internal standard (oleic acid), while "x" can be interpreted as the loading percentage of the analyte in the LDH/LDHc-BSE composites (see **Section 3.3.6** for details).

B) Parameters (mathematical equation and coefficient of determination) of the calibration curves for the absolute quantification of α-BA and β-BA (see **Section 3.3.6** for details).

A)

Analyte	Equation of the calibration curve	Coefficient of determination (R^2)
β-KBA	$y = (7.38 \cdot 10^{-3})\ x + 4.64 \cdot 10^{-3}$	0.9986
β-AKBA	$y = (2.67 \cdot 10^{-3})\ x + 6.75 \cdot 10^{-4}$	0.9997
BA isomer 3	$y = (2.24 \cdot 10^{-3})\ x + 1.81 \cdot 10^{-3}$	0.9994
BA isomer 4	$y = (4.34 \cdot 10^{-3})\ x + 1.03 \cdot 10^{-3}$	1.0000
BA isomer 5	$y = (2.12 \cdot 10^{-2})\ x + 1.03 \cdot 10^{-3}$	0.9997
BA isomer 6	$y = (-6.78 \cdot 10^{-6})\ x^2 + (2.14 \cdot 10^{-3})\ x + 8.42 \cdot 10^{-4}$	0.9999
α-BA	$y = (-2.5 \cdot 10^{-5})\ x^2 + (5.90 \cdot 10^{-3})\ x + 10^{-2}$	0.9964
β-BA	$y = (-4.46 \cdot 10^{-5})\ x^2 + (1.26 \cdot 10^{-2})\ x + 2.75 \cdot 10^{-2}$	0.9957
BA isomer 7	$y = (-5.81 \cdot 10^{-6})\ x^2 + (2.79 \cdot 10^{-3})\ x + 8.71 \cdot 10^{-4}$	0.9999
ABA isomer 1	$y = (1.39 \cdot 10^{-3})\ x + 1.03 \cdot 10^{-3}$	0.9987
ABA isomer 2	$y = (4.07 \cdot 10^{-4})\ x + 2.14 \cdot 10^{-4}$	0.9998
ABA isomer 3	$y = (3.37 \cdot 10^{-4})\ x + 3.40 \cdot 10^{-4}$	0.9977
ABA isomer 4	$y = (-1.55 \cdot 10^{-6})\ x^2 + (7.32 \cdot 10^{-4})\ x + 5.36 \cdot 10^{-5}$	1.0000
α-ABA	$y = (-6.91 \cdot 10^{-6})\ x^2 + (2.41 \cdot 10^{-3})\ x + 1.20 \cdot 10^{-3}$	0.9998
β-ABA	$y = (-2.82 \cdot 10^{-5})\ x^2 + (7.43 \cdot 10^{-3})\ x + 5.50 \cdot 10^{-3}$	0.9994

B)

Analyte	Equation of the calibration curve	Coefficient of determination (R^2)
α-BA	$y = (-3.01 \cdot 10^{-4})\ x^2 + (7.57 \cdot 10^{-2})\ x + 4.41 \cdot 10^{-2}$	0.9997
β-BA	$y = (-5.79 \cdot 10^{-5})\ x^2 + (1.12 \cdot 10^{-1})\ x + 4.32 \cdot 10^{-2}$	0.9998

Table S2.

A) Estimated percent loaded amount of boswellic acids in the LDH-BSE and LDHc-BSE composites. These values represent the percentage content of each analyte embedded in a given mass of LDH/LDHc-BSE composite, in respect to the amount that is enclosed in the same mass of BSE. The values are reported in the form of mean ± standard deviation (SD). Here, the mean and the SD refer to three extraction replicates performed on both the LDH-BSE and LDHc-BSE composite, following the extraction protocols described in **Section 3.3.5**.

B) Estimated loaded amount (μg/mg) of α-BA and β-BA in BSE, LDH-BSE and LDHc-BSE composites. In the latter two cases, the values are reported in the form of mean ± standard deviation (SD). Here, the mean and the SD refer to three extraction replicates performed on each inorganic-organic composite, following the protocols described in **Section 3.3.5**. In the case of BSE the value refers to the estimated α-BA and β-BA starting from the RPLC-ESI(-)-FTMS analysis of a methanolic solution (100 μg/mL) of lyophilized BSE.

A)

	Loaded amount (%)	
**Analyte**	**LDH-BSE**	**LDHc-BSE**
β-KBA	43 ± 6	45.0 ± 0.2
β-AKBA	41 ± 5	52.6 ± 1.7
BA isomer 3	45 ± 4	57.3 ± 1.9
BA isomer 4	45 ± 6	61.7 ± 0.8
BA isomer 5	50 ± 7	67 ± 4
BA isomer 6	53 ± 4	71 ± 8
α-BA	61 ± 11	105 ± 3
β-BA	63 ± 9	104 ± 2
BA isomer 7	44 ± 7	64.3 ± 1.6
ABA isomer 1	58 ± 10	80.4 ± 1.6
ABA isomer 2	70 ± 7	93 ± 8
ABA isomer 3	58 ± 9	77 ± 5
ABA isomer 4	62 ± 7	88.3 ± 1.3
α-ABA	58 ± 7	90 ± 3
β-ABA	56 ± 8	99.0 ± 0.4

B)

	Amount (μg/mg)		
**Analyte**	**LDH-BSE**	**LDHc-BSE**	**BSE**
α-BA	31 ± 3	42.8 ± 0.4	42
β-BA	55 ± 5	72.4 ± 0.9	74

Supplementary figures

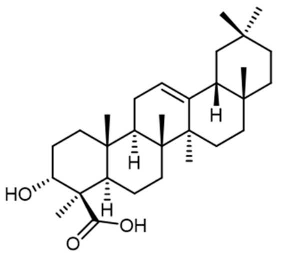

α-BA

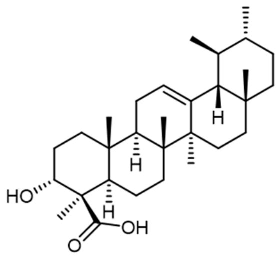

β-BA

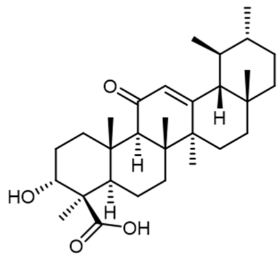

β-KBA

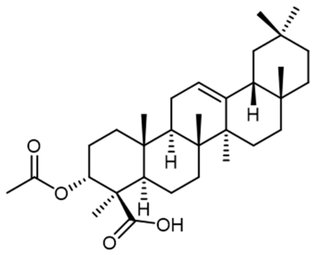

α-ABA

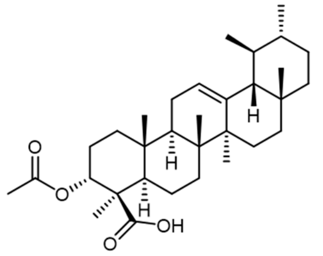

β-ABA

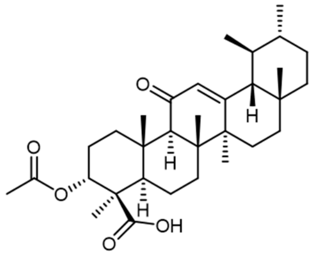

β-KABA

Figure S1.

Chemical structures of α-boswellic acid (α-BA), β-boswellic acid (β-BA), 11-keto-β-boswellic acid (β-KBA), 3-acetyl α-boswellic acid (α-ABA), 3-acetyl β-boswellic acid (β-ABA), and 3-acetyl 11-keto-β-boswellic acid (β-AKBA).

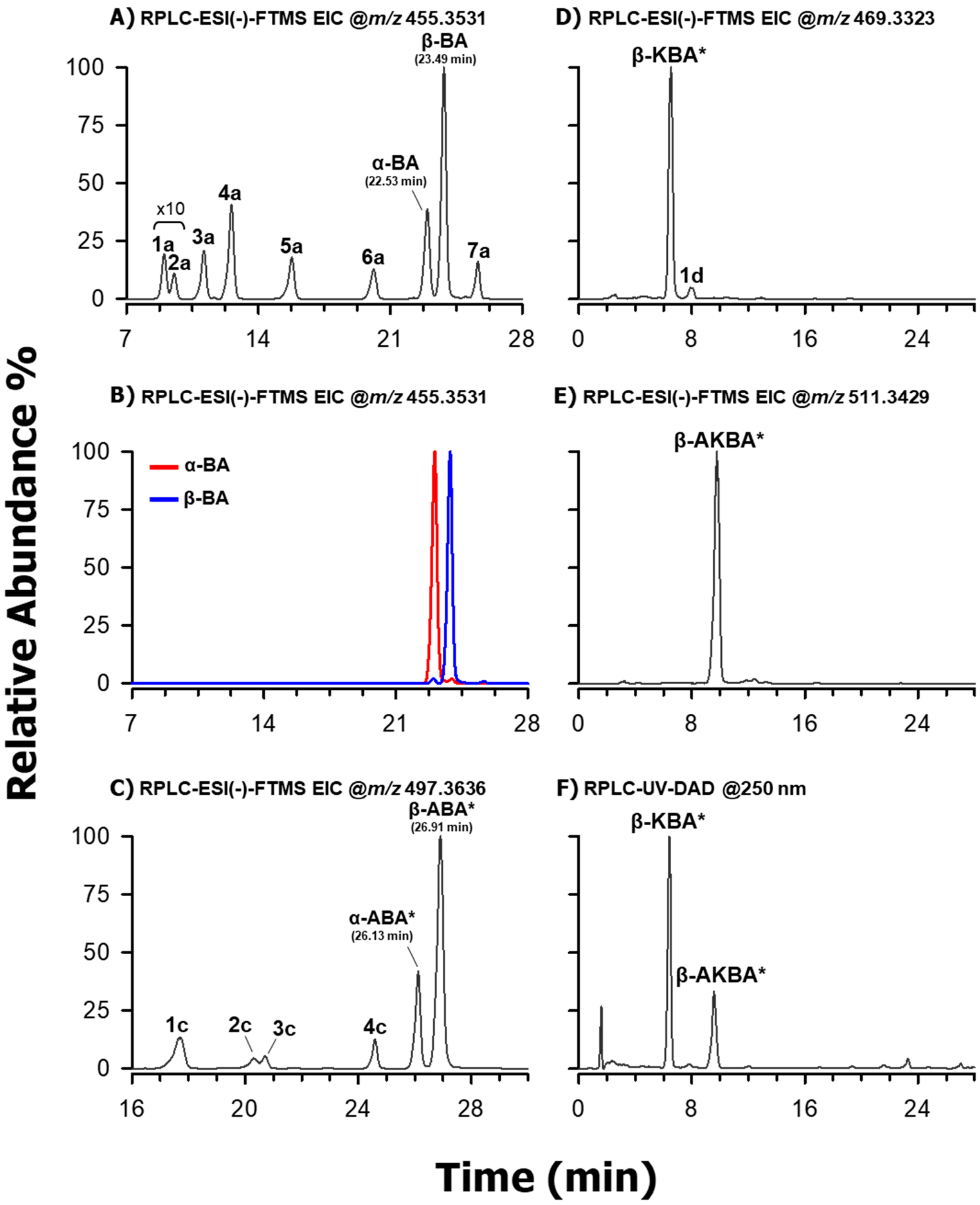

Figure S2.

Extracted ion chromatograms (EIC) referring to the RPLC-ESI(-)-FTMS analysis of a methanolic solution (100 µg/mL) of lyophilized BSE. The EIC traces were obtained by setting a 5 ppm extraction window centred on the theoretical *m/z* of the $[M-H]^-$ ions of (**A**) α-BA and β-BA (*m/z* 455.3531), (**C**) α-ABA, and β-ABA (497.3636), (**D**) β-KBA (469.3323), and (**E**) β-AKBA (*m/z* 511.3429).

Panel **B** displays the overlap of two EIC traces referring to the RPLC-ESI(-)-FTMS analysis performed on each of the two equally concentrated (10 µg/mL) methanolic solutions of the α-BA and β-BA analytical standards.

Panel **F** shows the RPLC-UV-DAD chromatogram recorded at 250 nm for a 1 mg/mL methanolic solution of lyophilized BSE.

The peak tags labelled with "*" refer to those species that were tentatively identified on the basis of the information emerging from experimental data (retention time, MS/MS and UV-Vis spectra) and previous literature studies (see the main text for details). For some peaks (*i.e.*, those corresponding to α-BA, β-BA, α-ABA, and β-ABA) the information about the retention time is also shown to support what stated in the main text.

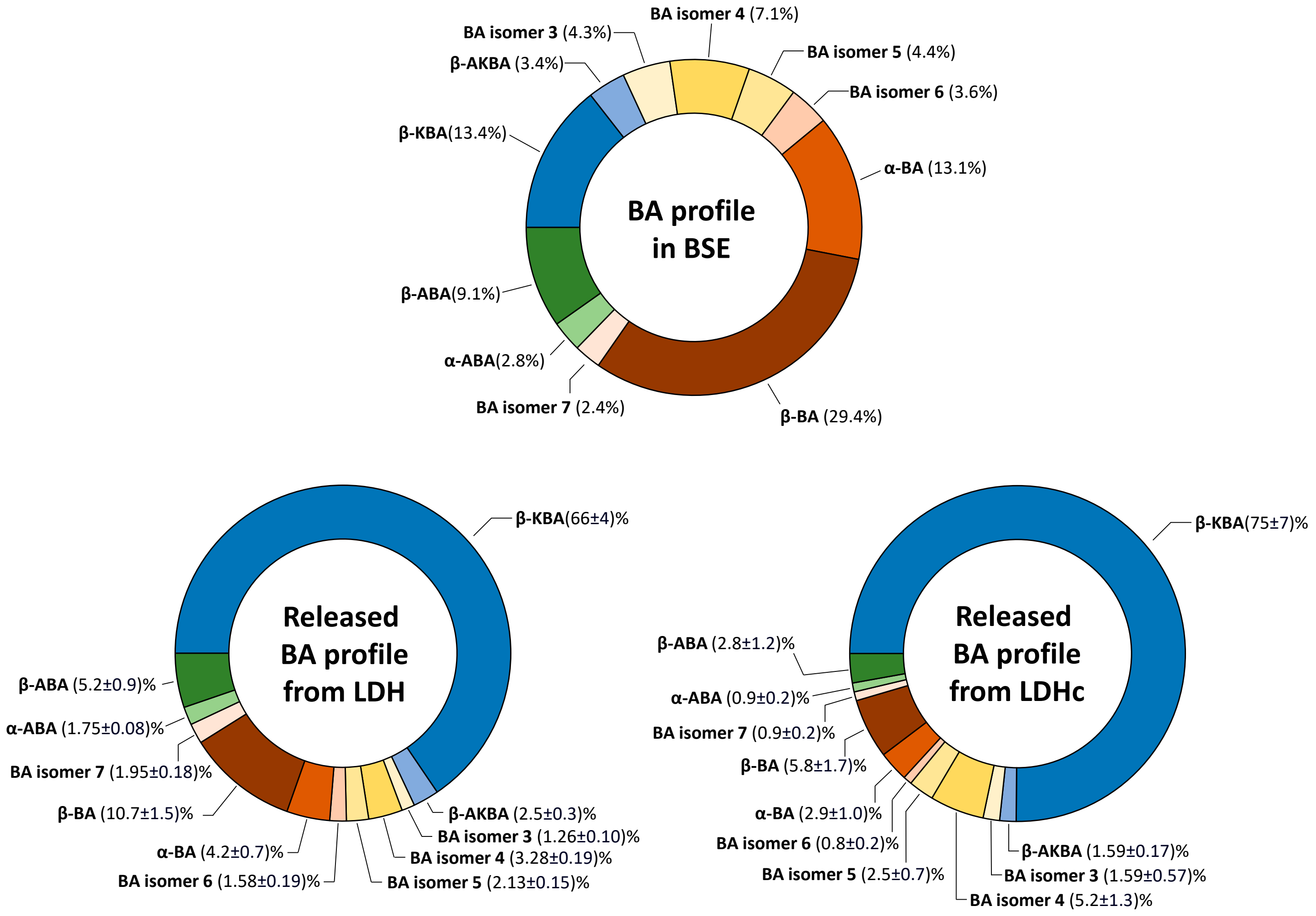

Figure S3.
Ring charts displaying the calculated BA profile in pure BSE and the profile of BA released in phosphate-buffered saline (PBS) solution from LDH-BSE and LDHc-BSE composites. The values indicate the percent ratio between the calculated EIC peak area of each BA and the sum of the EIC areas estimated for all the BA of interest (see **Section 2.4** for details). The values reported as mean ± standard deviation refer to triplicates of the release essay described in **Section 3.3.7**.

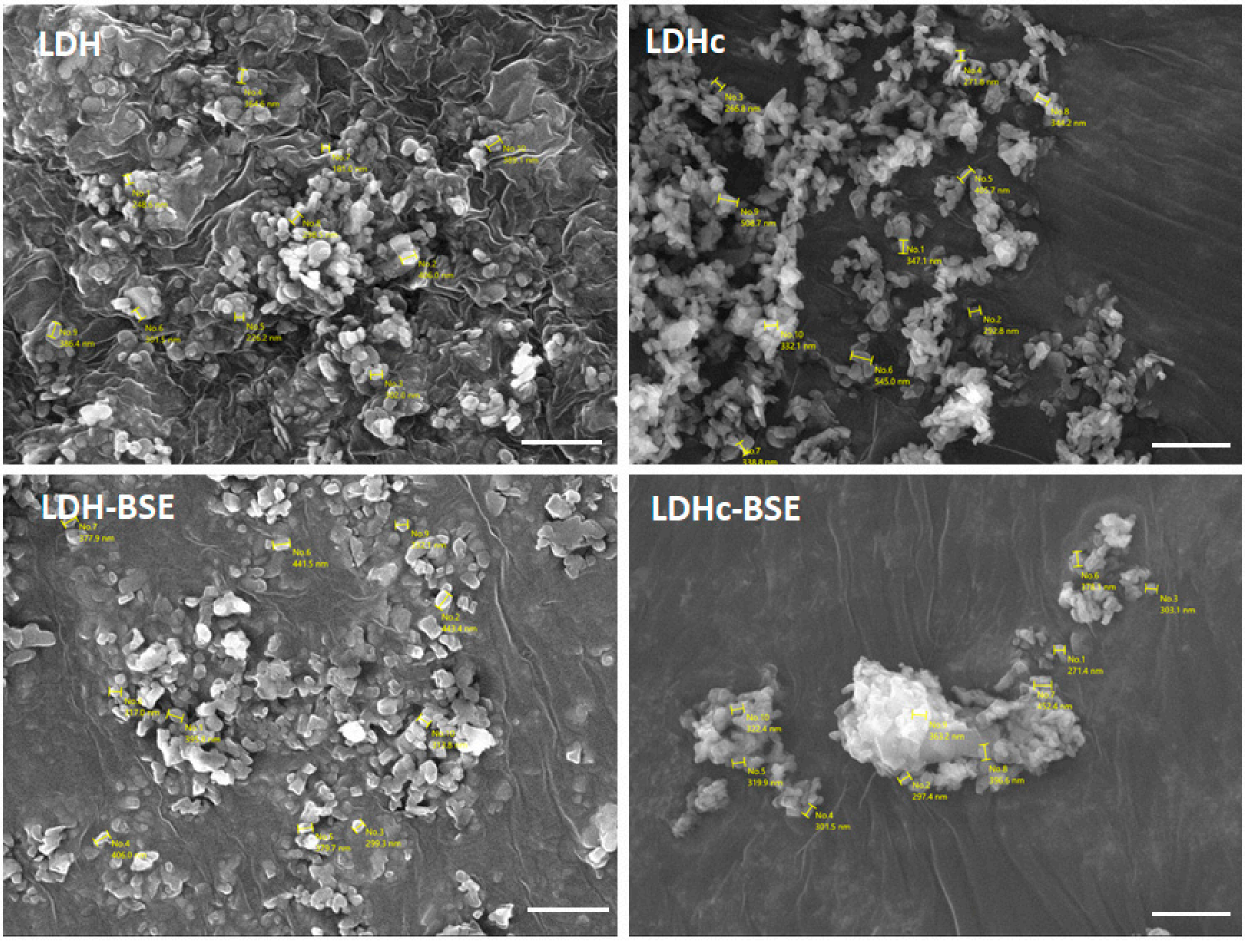

Figure S4.

SEM visual analysis of the developed composites' morphology. Images are representative for the diameter size estimation of the single nanocomposites randomly selected; calculation was obtained by the SEM software. Images magnification = 8.000x, bar scale = 2 µm.
